# Demographics and Characteristics of Cardiology Physician Assistants/Physician Associates in the United States: A Workforce Analysis

**DOI:** 10.1016/j.jacadv.2024.101394

**Published:** 2024-11-14

**Authors:** Sondra M. DePalma, Roderick S. Hooker, Mirela Bruza-Augatis, Andrzej Kozikowski, Kasey Puckett

**Affiliations:** a American Academy of Physician, Associates, Advocacy, Alexandria, Virginia, USA; b A.T. Still University Arizona School of Health Sciences Mesa, Arizona, USA; c Health Policy Analyst, Retired. Ridgefield, Washington, USA; d Research and Exams Department, Johns Creek, Georgia, USA

**Keywords:** cardiology, cardiovascular, demographics, physician assistant, physician associate, workforce

Physician assistants/physician associates (PAs) have emerged as strategic cardiology care team members, providing a diverse and vital role in cardiovascular disease (CVD) prevention and management.^1^ The PA cardiology workforce is expanding as the number of persons affected by CVD increases while the physician supply lags behind an optimal ratio for managing this burden.

A greater prevalence of CVD, along with emerging treatment technologies, is expected to increase the demand for cardiology services when there is a projected deficit of cardiovascular specialists relative to patient needs. Additionally, physicians specializing in CVD are among the oldest clinicians of all specialties, with 62.8% (over 14,000) of active cardiologists over 55 years in 2019.^2^ Predictably, many cardiologists will leave the workforce in the next 10 to 15 years, and the number of medical residents and fellows completing training will need to be increased to maintain the size of the cardiology workforce.^2^ This cardiologist shortage has led to the implementation of effective interventions to manage CVD, one of which is the implementation of team-based care that includes PAs.

Due to their extensive generalist medical training, PAs are well-equipped to provide high-quality, cost-effective CVD care. PAs are employed in the outpatient and inpatient settings, providing general and specialty cardiovascular care. The services PAs provide in inpatient cardiac care include history and physical examinations, consultations, discharge management services, hospital rounds, preprocedure and postprocedure management, stress testing, and cardiology procedures (eg, elective cardioversions, temporary pacing wires, and heart catheterizations). PAs in cardiology provide similar services in the outpatient setting, prescribe medications, manage cardiovascular implantable electronic devices, deliver patient education, and participate in cardiovascular research. Most of these services are performed autonomously without the direct involvement of a cardiologist.^1^

These services and this manner of PA practice lead to more effective and efficient cardiovascular care, which is crucial for improving patient outcomes. As PAs' impact in cardiology increases, their role and characteristics are essential for predictive modeling and staffing arrangements. Therefore, a workforce analysis was performed to understand their demographics, practice settings, and other factors to inform administrators, health care workforce analysts, researchers, and policymakers.

A quantitative study of PAs in cardiology was undertaken using the National Commission on Certification of PAs *PA Professional Profile* data set. This data set contains voluntary, self-reported demographic and practice information of board-certified PAs with an 83.9% participation rate in 2023. The *Sterling Institutional Review Board* (IRB#10826) deemed this study exempt.

The sample included a total count of 178,708 board-certified PAs in 2023. PAs who did not complete their PA profile within the last 3 years, reported they were not working clinically, or did not respond to the practice specialty question were excluded. Thus, the final study population was 126,941 board certified PAs. The analysis utilized descriptive statistics and chi-square or Mann-Whitney *U* tests to compare the demographic and practice attributes of PAs employed in cardiology to those in all other clinical specialties. SPSS, Version 29.0 (IBM Corp, Armonk, New York, USA) was used to perform all the statistical analyses. By the end of 2023, 3,705 (2.9%) board certified PAs were employed in cardiology.

PAs practicing in cardiology differed significantly in demographic characteristics from PAs in all other specialties in terms of gender, race, ethnicity, U.S. region, and urban-rural setting (all *P* < 0.05); however, no statistically significant differences were identified based on age (*P* = 0.089; Table 1). Among PAs who practice in cardiology, 61.8% reported working in hospital settings. PAs in cardiology report working slightly more hours per week (mean, SD) than those in all other specialties (41.0 [9.2] vs 39.7 [10.5]; *P* < 0.001), they see fewer patients per week (53.6 [29.0] vs 66.6 [41.8]; *P* < 0.001) and were more likely to hold one clinical position (88.5% vs 84.9%; *P* < 0.001). The percentage of PAs participating in telemedicine (*P* = 0.154) and years certified (*P* = 0.084) between PAs in cardiology and other specialties were similar (Table 1).

**Table 1:** Demographics, Practice, and Other Characteristics of PAs Practicing in Cardiology Compared to PAs in All Other Medical Specialties

	PAs in Cardiology (n = 3,705; 2.9%)	PAs in all Other Specialties (n = 123,236; 97.1%)	*P* Value
	Counts, N (%)	Counts, N (%)	
Demographic Characteristics			
Age, y			
Mean ± SD	40.8 ± 10.5	41.2 ± 10.9	0.089
Median (IQR)	38 (32-48)	39 (33-48)	
Sex			
Male	1,018 (27.5)	36,890 (29.9)	0.001
Female	2,686 (72.5)	86,318 (70.1)	
Race			
White	3,088 (86.0)	99,050 (84.0)	<0.001
Asian	249 (6.9)	7,812 (6.6)	
Black/African American	94 (2.6)	4,124 (3.5)	
Multirace	59 (1.6)	2,908 (2.5)	
Other races^a^	99 (2.8)	4,067 (3.4)	
Ethnicity			
Hispanic/Latino(a/x)	183 (5.1)	8,404 (7.1)	<0.001
Non-Hispanic/Latino(a/x)	3,404 (94.9)	110,220 (92.9)	
U.S. region			
Midwest	709 (19.2)	23,958 (19.5)	<0.001
Northeast	1,141 (30.8)	29,743 (24.2)	
South	1,305 (35.3)	42,667 (34.8)	
West	545 (14.7)	26,387 (21.5)	
Urban-rural setting			
Urban	3,528 (95.5)	113,447 (92.6)	<0.001
Rural/Isolated	165 (4.5)	9,057 (7.4)	
Practice Characteristics			
Practice setting			
Hospital	2,282 (61.8)	50,437 (41.1)	<0.001
Office-based private practice	1,177 (31.9)	45,725 (37.2)	
Federal government	104 (2.8)	5,650 (4.6)	
Other practices^b^	132 (3.6)	21,037 (17.1)	
Years certified in categories			
<10	1,892 (51.1)	63,581 (51.6)	0.250
11-20	1,086 (29.3)	36,793 (29.9)	
21+	727 (19.6)	22,862 (18.6)	
Hours worked per week			
Mean ± SD	41.0 ± 9.2	39.7 ± 10.5	<0.001
Median (IQR)	40 (40-45)	40 (36-45)	
Patients seen each week			
Mean ± SD	53.6 ± 29.0	66.6 ± 41.8	<0.001
Median (IQR)	50 (35-66.5)	60 (40-88)	
Secondary position			
No, I work in only 1 clinical position	3,230 (88.5)	103,142 (84.9)	<0.001
Yes, I also work in a position where I do not provide direct patient care (ie, education, research, administration)	108 (3.0)	4,331 (3.6)	
Yes, I work in 2 or more clinical PA position	310 (8.5)	13,985 (11.5)	
Participate in telemedicine			
No	2,074 (56.2)	70,534 (57.4)	0.154
Yes	1,618 (43.8)	52,450 (42.6)	
Income			
Mean ± SD	$121,052 ± $29,427	$124,065 ± $35,408	<0.001
Median (IQR)	$115,000 ($105,000-$135,000)	$125,000 ($105,000-$145,000)	
Job satisfaction of current job			
Not satisfied^c^	543 (15.1)	20,329 (17.0)	0.003
Satisfied^d^	3,051 (84.9)	99,204 (83.0)	
Burnout			
No symptoms of burnout^e^	2,439 (68.6)	77,691 (65.7)	<0.001
One or more symptoms of burnout^f^	1,117 (31.4)	40,493 (34.3)	
Plan to leave principal clinical position in next 12 mo			
No	3,348 (91.6)	110,124 (90.6)	0.045
Yes	306 (8.4)	11,360 (9.4)	
Plan to retire in the next 5 y			
No	3,491 (94.7)	115,487 (94.0)	0.079
Yes	194 (5.3)	7,316 (6.0)	

Values are mean ± SD, median (IQR), or n (%).

PA = physician assistant/physician associate.

a Other races include those who selected "other," Native Hawaiian/Pacific Islander, and American Indian/Alaska Native.

b Other practices include behavioral/mental health facilities, community health centers, nursing homes, occupational health settings, school- or college-based health centers, or school clinics.

c Not satisfied includes "neither satisfied nor dissatisfied," "somewhat dissatisfied," "mostly dissatisfied," and "completely dissatisfied."

d Satisfied includes "completely satisfied," "mostly satisfied," and "somewhat satisfied."

e No symptoms of burnout include “I enjoy my work; I have no symptoms of burnout,” “Occasionally, I am under stress, and I don't always have as much energy as I once did, but I don’t feel burned out.”

f One or more symptoms of burnout include "I am definitely burning out and have one or more symptoms of burnout, such as physical and emotional exhaustion." "The symptoms of burnout that I'm experiencing won't go away. I think about frustration at work a lot," and " I feel completely burned out and often wonder if I can go on. I am at the point where I may need some changes or may need to seek some sort of help."

Table 1 also displays job satisfaction, burnout, plans to leave current clinical position, and retirement plans of PAs in cardiology versus PAs in all other medical specialties. Among the 3,705 PAs in cardiology, a slightly higher proportion (84.9%) reported satisfaction with their job compared with 83.0% of other specialties (*P* = 0.003). Moreover, PAs in cardiology were also more likely to report no symptoms of burnout (68.6% vs 65.7%; *P* < 0.001) and less likely to report that they had intentions to depart from their principal clinical position within 12 months (8.4% vs 9.4%; *P* = 0.045). Both PA cohorts had similar rates of planning to retire in the next 5 years (5.3% vs 6.0%; *P* = 0.079).

This study found that of the 2.9% of PAs practicing in cardiology, almost three-quarters (72.5%) are female, compared to less than 15% of physician cardiologists in the United States.^3^ This contrast is important because provider diversity, including gender, may be critical to reducing health care disparities. Furthermore, demographic concordance between patients and practitioners is associated with patient trust and satisfaction, patient-centered communication, and guideline-directed medical management.^4^^,^^5^ The increased inclusion of PAs in cardiology could offset the gender disparity among practitioners within the specialty and potentially improve health care delivery and outcomes. Compared to clinically active cardiologists, PAs in cardiology are considerably younger. As of 2019, 62.8% of active cardiologists were over 55 years old;^2^ in 2023, only 20.8% of PAs in cardiology were 50 years old or older, and only 5.3% plan to retire in the next 5 years. Thus, this PA workforce should remain in clinical practice longer than many cardiologists, which could blunt the projected cardiovascular workforce deficit.

There are several limitations to the study and data. The *PA Professional Profile* is composed of voluntary input and is subject to social desirability biases and nonresponse errors. Although some demographic characteristics between cardiology and noncardiology PAs were statistically significant, the large number of respondents may have led to statistical significance when the absolute differences were slight. While this study includes data on demographics and some practice characteristics, the subspecialties of cardiology PAs and the specific services performed by PAs in cardiology were not assessed. Also, data are limited in that information about other practitioners (eg, advanced practice registered nurses) was not available.

In conclusion, in 2023, 3,705 board-certified PAs (2.9%) were employed in a cardiology setting. Most PAs in cardiology identify as female (72.5%) and report a median age of 38. Most reside in an urban setting (95.5%), and 61.8% practice in a hospital. Understanding the demographics and practice characteristics of a critical arm of the cardiology workforce is essential as team-based care grows to meet the needs of an increasing and aging population.

## Funding support and author disclosures

The authors have reported that they have no relationships relevant to the contents of this paper to disclose.
